# Psychological impact of the 2023 Kahramanmaraş earthquakes: a systematic review and meta-analysis of PTSD, depression, and anxiety among Turkish adults

**DOI:** 10.3389/fpubh.2025.1664212

**Published:** 2025-08-26

**Authors:** Metin Çınaroğlu, Eda Yılmazer, Esra Noyan Ahlatcıoğlu, Selami Varol Ülker, Gökben Hızlı Sayar

**Affiliations:** ^1^Psychology Department, İstanbul Nişantaşı University, İstanbul, Türkiye; ^2^Psychology Department, Beykoz University, İstanbul, Türkiye; ^3^İstanbul Provincial Health Directory, İstanbul, Türkiye; ^4^Psychology Department, Üsküdar University, İstanbul, Türkiye; ^5^Medical School, Üsküdar University, İstanbul, Türkiye

**Keywords:** Kahramanmaraş earthquakes, Türkiye, post-traumatic stress disorder (PTSD), depression, anxiety, earthquake survivors, disaster mental health

## Abstract

**Background:**

The twin earthquakes that struck Kahramanmaraş, Türkiye, on February 6, 2023, caused widespread devastation and loss of life. Beyond the physical destruction, such large-scale disasters often result in significant psychological trauma. This study systematically reviewed and meta-analyzed the prevalence and severity of probable post-traumatic stress disorder (PTSD), depression, and anxiety among adult Turkish survivors during the first 18 months post-disaster.

**Methods:**

Following PRISMA 2020 guidelines, a systematic search of Web of Science, PubMed, and Scopus was conducted for peer-reviewed studies published between February 6, 2023, and May 30, 2025. Eligible studies included quantitative assessments of PTSD, depression, or anxiety using validated Turkish-language scales, with general adult population samples (*N* ≥ 370). Eight studies (*N* = 5,965) met inclusion criteria. A random-effects meta-analysis was conducted for studies reporting prevalence of probable PTSD, while depression and anxiety outcomes were synthesized descriptively due to limited and heterogeneous data. Risk factors for psychological morbidity were also extracted and analyzed. This review was registered with PROSPERO (CRD42025644127).

**Results:**

The pooled prevalence of probable PTSD was 41% (95% CI: 32–52%). Reported PTSD rates ranged from 29 to 54%, and symptom severity remained high throughout the first year. Depression and anxiety were also widespread, with up to 40% screening positive for depression and 40–50% reporting moderate-to-severe anxiety symptoms. Comorbidity between PTSD, depression, and anxiety was common. Significant risk factors included female gender, bereavement, home destruction, displacement, job loss, and low social support. Resilience was protective in some studies, though findings were inconsistent.

**Conclusion:**

Eighteen months after the 2023 Kahramanmaraş earthquakes, Turkish adult survivors continued to experience high levels of probable PTSD, depression, and anxiety. These findings highlight a prolonged mental health crisis and underscore the urgent need for sustained, targeted psychosocial interventions. Integrating mental health support into disaster preparedness and long-term recovery efforts is essential for mitigating psychiatric morbidity in future disasters.

**Systematic review registration:**

https://www.crd.york.ac.uk/PROSPERO/view/CRD42025644127.

## 1 Introduction

The twin earthquakes of February 6, 2023 in Kahramanmaraş, Türkiye – of magnitudes 7.7 and 7.6 – constituted one of the most devastating natural disasters in the country’s history, causing over 50,000 deaths and massive destruction across 11 provinces (1). Beyond the immense physical toll, such large-scale earthquakes are known to precipitate severe and long-term psychological effects in survivors (2). Prior research on major earthquakes worldwide has documented elevated rates of PTSD (3), depression, and anxiety in affected populations (4). For instance, a meta-analysis of 46 studies reported an average PTSD prevalence of ~23.7% among earthquake survivors, with higher combined incidence (~28.8%) when assessed within 9 months post-disaster (5). However, PTSD rates vary widely across events (ranging from ~1 to >80% in individual studies) depending on disaster severity and sample characteristics (6–8). Other disorders such as depression and generalized anxiety also commonly increase after earthquakes (9, 10) – e.g. one post-earthquake survey in Peru found 52% with anxiety and 52% with depression symptoms 1 month after the event (11, 12).

Past Turkish earthquakes provide relevant context. Following the 1999 Marmara earthquake, estimated PTSD prevalence in survivors was about 25–43% at 1 year and ~23% at 14 months (13, 14). These figures, while substantial, may be eclipsed by the psychological impact of the 2023 Kahramanmaraş earthquakes given their unprecedented scale. Early reports predicted that the trauma from the 2023 quakes would be profound and potentially long-lasting (15, 16). Indeed, preliminary studies shortly after the disaster indicated extremely high acute stress levels – one survey 50 days post-quake found that roughly 69% of adult survivors scored above the clinical cutoff for PTSD symptoms (17). This raises urgent questions about the prevalence of PTSD and other mental health problems as the affected communities progress through the first year of recovery. Rigorous synthesis of emerging evidence is needed to quantify the psychological toll and identify factors associated with worse outcomes.

We hypothesize that the psychiatric impact of the 2023 Kahramanmaraş earthquakes may exceed that observed in prior Turkish and international disasters for several interrelated reasons. First, the magnitude and scope of destruction—with twin quakes, widespread infrastructure collapse, and over 50,000 fatalities—was unprecedented in recent Turkish history. Second, the scale of displacement and prolonged housing instability, with hundreds of thousands still in temporary shelters more than a year later, may have exacerbated chronic stress. Third, the disaster occurred in a context of ongoing socioeconomic challenges, including inflation and limited mental health infrastructure in the affected provinces. Finally, the cumulative trauma experienced by survivors (e.g., bereavement, injury, entrapment, repeated aftershocks) likely contributed to elevated psychiatric comorbidity. These factors, individually and collectively, may have intensified the severity and persistence of psychological distress in the aftermath of the 2023 earthquakes.

## 2 Methods

### 2.1 Study design

This study is a systematic review and meta-analysis designed to evaluate the psychological impact of the 2023 Kahramanmaraş earthquakes on Turkish adult survivors. It was developed in accordance with the PRISMA 2020 guidelines. The protocol was registered with the International Prospective Register of Systematic Reviews (PROSPERO) under the registration number CRD42025644127.

The aim of the study was to synthesize and quantify the prevalence and severity of PTSD, depression, and anxiety in the general adult population directly affected by the earthquakes. The review focused exclusively on peer-reviewed quantitative studies using standardized and culturally validated Turkish versions of internationally recognized psychological assessment tools. Only studies examining the general adult population were considered; those targeting specific subgroups (e.g., children, students, refugees, or occupational groups) were excluded to ensure sample homogeneity and generalizability.

The review focused exclusively on peer-reviewed quantitative studies using standardized and culturally validated Turkish versions of internationally recognized psychological assessment tools. Psychological outcomes in the included studies were assessed using Turkish-validated versions of established instruments. PTSD symptoms were measured using the PTSD Checklist for DSM-5 (PCL-5; Cronbach’s *α* = 0.94), the Clinician-Administered PTSD Scale for DSM-5 (CAPS-5; *α* > 0.90), and the International Trauma Questionnaire (ITQ; PTSD scale *α* = 0.91, DSO scale *α* = 0.87). Depression was assessed using the Beck Depression Inventory-II (BDI-II; *α* = 0.90 non-clinical, *α* = 0.89 clinical), and anxiety was assessed with the Beck Anxiety Inventory (BAI; *α* = 0.93), which also showed high internal consistency in Turkish populations.

### 2.2 Search strategy

A comprehensive and systematic search of the literature was undertaken to identify relevant studies examining the psychological effects of the 2023 Kahramanmaraş earthquakes on Turkish adult populations. The strategy was developed in alignment with PRISMA 2020 guidelines to ensure transparency, reproducibility, and methodological rigor.

Three academic databases—WOS, PubMed, and Scopus—were systematically searched for peer-reviewed journal articles published between February 6, 2023, and May 30, 2025. This time window was selected to capture studies conducted and disseminated within the acute and subacute aftermath of the earthquakes. In addition to database searches, reference lists of included studies were manually reviewed to identify any further eligible publications.

The search terms were developed through an iterative process and included combinations of keywords and subject terms related to the disaster event (such as “Kahramanmaraş earthquake,” “2023 Turkey earthquake,” “Türkiye earthquake survivors”), the population of interest (“earthquake survivors,” “disaster-affected adults”), and psychological outcomes (“PTSD,” “post-traumatic stress,” “depression,” “anxiety,” “mental health”). Boolean operators were used to structure the queries and refine the results. Filters were applied to limit retrieval to studies published in English, conducted with human participants, and published in peer-reviewed outlets (for more details, see Supplementary material).

The initial search strategy was drafted by E. Y. and E. N. A., with revisions and refinement provided by S. V.Ü. And M.Ç. supervised the development of the final version, and the full database search was independently executed by G. H. S. in May 2025. All retrieved records were exported into a reference management system, and duplicates were removed through both automated and manual screening prior to the study selection phase.

### 2.3 Study selection

Following the database search and deduplication process, all records were screened for eligibility in two stages: title/abstract review and full-text assessment. Eligibility was determined based on predefined inclusion and exclusion criteria, as outlined in the PROSPERO-registered protocol.

In the first stage, two reviewers (E. Y. and E. N. A.) independently screened the titles and abstracts of all retrieved records to identify potentially relevant studies. Articles that clearly did not meet inclusion criteria—such as those focusing on non-Turkish populations, qualitative studies, published none WOS, Scopus or Pubmed indexed journals, editorials, published not in English, or unrelated psychological topics—were excluded at this stage. Studies that appeared potentially eligible were advanced to full-text review.

In the second stage, full-text articles were retrieved and assessed in detail for eligibility by S. V.Ü. and M.Ç. Discrepancies between reviewers were resolved through discussion, and where necessary, consultation with G. H. S. served as the final arbiter. Inclusion was restricted to peer-reviewed quantitative studies that assessed PTSD, depression, or anxiety in adult Turkish survivors of the 2023 Kahramanmaraş earthquakes, using standardized and validated Turkish-language instruments. Studies focusing on special populations (e.g., children, students, healthcare workers, refugees) or those with sample sizes under 370 were excluded. To ensure robust and generalizable estimates, we included only studies with a minimum sample size of *N* ≥ 370, which approximates the required size to estimate prevalence with 95% confidence and ±5% precision under conservative assumptions (*p* ≈ 0.50).

**Figure 1:**
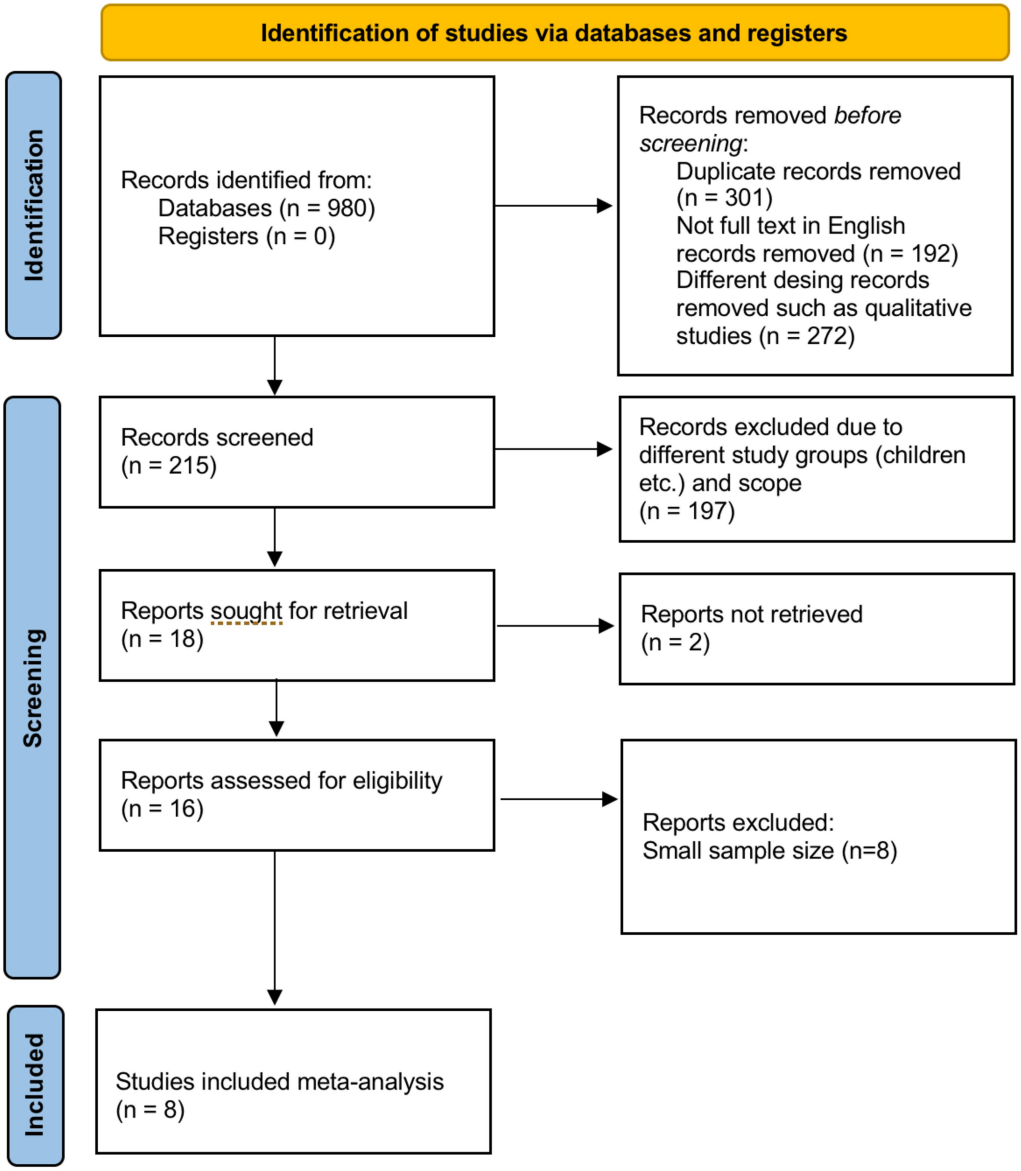
PRISMA flow chart.

Out of an initial pool of 980 records, 215 articles were evaluated at full text. Eight studies met all inclusion criteria (18–25) and were retained for data extraction and synthesis. Reasons for full-text exclusion were recorded and are presented in the PRISMA flow diagram (Figure 1).

### 2.4 Data extraction

A structured data extraction process was implemented to collect the relevant variables needed for synthesis and meta-analysis. Extraction focused on methodological characteristics, sample details, and psychological outcome data related to PTSD, depression, and anxiety.

Two authors (E. Y. and E. N. A.) independently extracted information from the final set of included studies using a standardized form. Extracted variables included sample size, population demographics, geographic location, timing of assessment, and study setting. Measurement instruments for psychological outcomes were recorded along with scoring methods, clinical cutoffs (if reported), and reporting format (e.g., prevalence rates, mean scores). Where applicable, data on subgroup comparisons and correlates such as gender, trauma exposure, or displacement were also noted.

All extracted data were reviewed for consistency and accuracy by S. V.Ü. and M.Ç. Disagreements were resolved by discussion, and G. H. S. provided oversight for methodological alignment. Finalized data were compiled into structured spreadsheets to support both the narrative synthesis and statistical analyses.

### 2.5 Quality and bias assessment

The methodological quality and potential sources of bias in the included studies were assessed using a structured, domain-based approach appropriate for observational designs. The assessment focused on factors such as sample representativeness, use of validated outcome measures, clarity in reporting, and the completeness of outcome data.

Each study was independently evaluated by E. Y., E. N. A. and M.Ç. using a standardized checklist adapted from the National Institutes of Health (NIH) Quality Assessment Tool for Observational Cohort and Cross-Sectional Studies. Criteria included adequacy of sample size, transparency of inclusion criteria, appropriateness of the measurement tools for PTSD, depression, and anxiety, and whether statistical analyses addressed potential confounders.

Studies were not excluded based on quality scores; rather, assessments were used to contextualize findings and inform sensitivity considerations during synthesis. Disagreements in ratings were resolved through discussion, with arbitration by GHS when necessary. The overall quality of included studies was rated as moderate to high. Most used well-validated instruments and sampled from relevant earthquake-affected regions, although several relied on convenience sampling, which may limit generalizability.

Risk of reporting bias across studies (e.g., publication bias) was considered in the interpretation of pooled results but was not formally tested due to the limited number of studies included in the meta-analysis.

A detailed summary of quality ratings for each included study is presented in Supplementary material. Each study was evaluated across the NIH tool’s criteria for observational cohort and cross-sectional designs, including sample representativeness, measurement validity, outcome completeness, and confounding control. Overall, five studies were rated as moderate quality (meeting 6–7 of 9 criteria), and three were rated as high quality (meeting 8–9 of 9 criteria). Common limitations included reliance on convenience sampling and limited reporting on non-responders. These quality assessments informed the interpretation of results but did not lead to exclusion of any studies.

### 2.6 Statistical analyses

Quantitative synthesis was performed for PTSD outcomes, which were consistently reported across the included studies. Meta-analytic procedures were conducted using a random-effects model to account for expected heterogeneity across study populations, instruments, and settings. Prevalence estimates of probable PTSD were extracted and pooled using the DerSimonian–Laird method. To stabilize variance and accommodate proportional data, prevalence rates were transformed using the Freeman–Tukey double arcsine transformation prior to aggregation and were back-transformed for interpretability. Mean PTSD scores were also descriptively summarized when prevalence data were not available. Due to variability in reporting and limited comparable data, depression and anxiety outcomes were synthesized narratively and via descriptive statistics.

Statistical heterogeneity was assessed using the Q statistic and the I^2^ index. High heterogeneity (*I*^2^ > 75%) was anticipated given differences in sampling frames, assessment timepoints, and geographic exposure levels. Where feasible, subgroup analyses were conducted to explore the effects of timing of assessment (early vs. late post-disaster) and study setting (e.g., displaced vs. community-based survivors). Sensitivity analyses were planned to test the influence of individual studies on pooled estimates but were constrained by the limited number of eligible studies.

All statistical procedures were performed using SPSS (version 30) and Python (version 13.3.1), with meta-analytic coding and visualizations prepared by S. V.Ü., E. N. A. and reviewed by M.Ç. and E. Y. A two-tailed *p*-value < 0.05 was considered statistically significant for all analyses.

## 3 Results

### 3.1 Study characteristics

Table 1 summarizes the key characteristics of the eight included studies. In total, the studies comprised 5,965 earthquake survivors (individual study sample sizes ranged from 383 to 2,034). All studies were conducted in Türkiye and published between mid-2023 and mid-2025, reflecting data collected within 18 months after the February 2023 earthquakes. Six of the eight studies [focused on survivors drawn from the hardest-hit provinces (e.g., Kahramanmaraş, Hatay, Adıyaman, Malatya)] or large clusters of displaced survivors, while two studies surveyed survivors who had relocated to other regions (e.g., to Mersin). The timing of assessments varied: two studies collected data within ~2–3 months post-earthquake, three studies at ~6–9 months post-disaster, and three studies around the 12–18 month mark. All studies employed cross-sectional survey designs using validated self-report measures.

**Table 1:** Included studies of psychological outcomes after the 2023 Kahramanmaraş earthquakes.

Study (first author, year)	Sample (N)	Timing post-quake	Setting/population	PTSD measure	Depression measure	Anxiety measure
İlhan et al. (22)	383 adult survivors (ED patients)	~3 months (May 2023)	Emergency dept. of tertiary hospital (Kırıkkale); mixed-gender	PCL-5 (Turkish) – PTSD Checklist DSM-5; cutoff for “probable PTSD” applied	None (PTSD-focused)	None
Alpay et al. (25)	527 adults (convenience sample)	~1.5–2.5 months (Mar–Apr 2023)	Community survivors in Mersin (many displaced from quake region)	ITQ (ICD-11 PTSD) – symptom clusters and diagnosis criteria; prevalence of probable PTSD reported	None	None
Yılmaz and Erdem (19)	400 adults in temporary shelters	~4 months (June 2023)	Tent camps in Hatay province (central districts); cluster sample	PCL-5 (Turkish) – PTSD Checklist DSM-5; cutoff applied for prevalence	BDI-II (Turkish) – Beck Depression Inventory; cutoff ~17 for depression	None
Akçay et al. (18)	2,034 adults across 11 provinces	~2–4 months (Mar–May 2023)	Multi-region survey (hard-hit provinces); convenience via online and field outreach	“Post-Earthquake Trauma Level Determination Scale” (64) – measures PTSD-like trauma symptoms; threshold defined for high vs. low trauma	None (GHQ-12 used as distress indicator in a related analysis)	None
Kıymış and Fakiroğlu (20)	662 adult survivors (multi-site)	~6 months (July–Aug 2023)	Communities across 9 affected provinces; volunteer sample	CAPS-5 (Clinician-Administered PTSD Scale) – used as 20-item symptom scale (0–4); analyzed as PTSD severity in SEM (no binary diagnosis)	PROMIS Depression 8-item short form (Turkish) – Likert 1–5; analyzed as continuous score	PROMIS Anxiety 7-item short form – Likert 1–5; continuous score
Filazoğlu Çokluk et al. (21)	923 adults in temporary shelters (Antakya)	~9 months (Oct 2023)	Tent city in Antakya, Hatay; all had proximal loss (lost close persons)	PCL-5 (Turkish) – PTSD Checklist DSM-5; continuous scores (mean/SD); regression for PTSD symptoms	BDI-II (Turkish); continuous (mean/SD)	BAI (Turkish); continuous (mean/SD)
Kaya et al. (24)	412 adults in one camp (Hatay)	~12 months (late Jan 2024)	Temporary container camp in Hatay; door-to-door survey of all eligible	PDS (Posttraumatic Diagnostic Scale, Turkish) – yields PTSD severity and category; “moderate” and “severe” PTSD rates reported	Brief Resilience Scale (for resilience; depression not measured)	None (anxiety not measured)
Taşkın et al. (23)	624 adults (mixed gender)	~18 months (Feb 2024)	General community in Kahramanmaraş city; recruited by outreach 1 year later	PCL-C/PCL-S (17-item PTSD Checklist, Turkish) – focusing on index event; continuous (mean/SD) and probable PTSD % (not explicitly reported, but analyzed)	None	None

ED, emergency department; SEM, structural equation modeling.

#### 3.1.1 PTSD measures

Each study assessed PTSD symptoms using a standardized instrument. Four studies used the PTSD Checklist (PCL) or its variants: two employed the DSM-5 version (PCL-5), one used the DSM-IV civilian version (PCL-C/PCL-S, 17-item), and one used the International Trauma Questionnaire (ITQ) for ICD-11 PTSD criteria. Another study applied the Primary Care PTSD Screen (PC-PTSD-5) as a screening tool, and one study used the Post-Traumatic Diagnostic Scale (PDS). All PTSD scales were validated Turkish versions; for instance, the ITQ was used to determine ICD-11 PTSD diagnosis in one study, and the clinician-administered CAPS-5 (Turkish) was referenced as the PTSD measure in another (though effectively used as a self-report analog in analysis). PTSD symptom severity was typically reported either as mean total scores or as prevalence of *probable PTSD* based on established cutoff scores (e.g., PCL-5 cutoff ~31–33 for probable PTSD, PCL-C cutoff ~44, PC-PTSD threshold of 3 “yes” responses).

#### 3.1.2 Depression and anxiety measures

Fewer studies evaluated depression or anxiety explicitly. Three studies administered the Beck Depression Inventory (BDI), and two of these also administered the Beck Anxiety Inventory (BAI). One large-scale study used the Adult Anxiety and Depression Short Forms (7-item and 8-item scales, respectively) from Patient-Reported Outcomes Measurement Information System (PROMIS), each scored 1–5, to quantify anxiety and depression levels in the sample. Another study utilized the General Health Questionnaire-12 (GHQ-12) as a broad measure of psychological distress, though this instrument does not separate depression/anxiety by subscale. All instruments were previously validated in Turkish populations.

Across the studies, participants were adults ages ~18 to 70+ (with mean ages in the 30s or 40s in most samples) and included somewhat more women than men overall (ranging from ~49 to 75% female in different studies; in the combined sample roughly 55% were female). All participants were directly exposed to the earthquakes – e.g., being present in the earthquake zone during or immediately after the events – and many endured significant trauma such as loss of family members, physical injuries, entrapment under rubble, destruction of homes, and displacement to temporary accommodations. Indeed, in the studies that reported exposure details, a majority of survivors had experienced multiple trauma types due to the earthquake. For example, Alpay et al. (25) noted that 99.2% of their sample experienced at least one quake-related traumatic event and 80.7% experienced four or more such events.

All included studies were rated as moderate-to-high quality in terms of using standard measures and clearly defining PTSD outcomes. A notable limitation in several was the use of convenience sampling (e.g., surveying those in camps or those presenting to an emergency department), which might skew prevalence estimates if, for instance, those with the most severe symptoms are more or less likely to participate. Despite this, the large sample sizes and consistency of findings across different settings lend confidence in the overall trends identified.

As shown in Table 1, five studies reported PTSD prevalence (as a percentage meeting a criteria threshold), while the others provided continuous PTSD severity scores. Fewer studies provided prevalence figures for depression or anxiety, though several reported mean scores. Despite methodological differences, a consistent picture emerges of a very high psychological burden in the surveyed survivor populations, as detailed below.

### 3.2 PTSD prevalence and severity

All eight studies found substantial levels of PTSD symptoms among survivors, with many individuals exceeding clinical screening thresholds for probable PTSD. Reported PTSD prevalence rates ranged from approximately 29–54% of participants across studies. The lowest PTSD prevalence was 29.0%, observed in a population-based survey of survivors in Hatay at 4 months post-quake (using PCL-5). In contrast, the highest PTSD prevalence was 54.1%, reported by Alpay et al. (25) in a sample of adults surveyed ~2 months post-quake in Mersin (using ICD-11 criteria via the ITQ). Several other studies found intermediate prevalence: for example, İlhan et al. (22) reported 51.4% of their sample had *probable PTSD* about 3 months after the disaster (using PCL-5), and Kaya et al. (24) found 36.2% of camp residents met criteria for at least moderate-to-severe PTSD ~1 year post-disaster (using the PDS). Despite some variability, these figures all indicate that roughly one-third to one-half of exposed adults were experiencing clinically significant PTSD symptoms in the months following the earthquakes.

We pooled the PTSD prevalence data using a random-effects model. The pooled prevalence of PTSD among survivors was approximately 41% (95% confidence interval [CI] ≈ 32–52%). This meta-analytic estimate implies that about two out of every five directly exposed adults met symptom criteria for PTSD in the aftermath of the 2023 earthquakes. Statistical heterogeneity was very high (*Q*-test *p* < 0.001, *I*^2^ ~ 95%), reflecting the range of estimates and differences in study methods/samples. We explored potential reasons for heterogeneity through subgroup analyses:

#### 3.2.1 By assessment timing

Studies conducted within the first 3 months tended to report higher PTSD rates (around 50% or more), whereas one study at 4 months found a lower rate (29%). However, by 12–18 months post-quake, PTSD prevalence remained markedly elevated: Taskın et al. (23) did not report a percentage, but noted a mean PTSD score of 57.9 (SD 14.9) on the PCL (out of 85) 1 year later, a level consistent with a high PTSD rate (in fact, higher than the ~40% at 1 year observed after the 2008 Wenchuan earthquake) (25). Similarly, Kaya et al. (24) at 12 months found 36% with moderate/severe PTSD, indicating no dramatic drop-off in PTSD prevalence between 4 months and 1 year in that hard-hit camp sample. In summary, PTSD prevalence did not uniformly decline over the first year; if anything, substantial proportions of survivors continued to report PTSD symptoms at 9–12 months comparable to the acute phase. Immediate post-disaster studies (within ~6 weeks) were limited, but an initial small study (~50 days post-quake) found 69% above the PTSD cutoff (17), suggesting an extremely acute spike which may have modestly tempered by 4–6 months (to ~30–50%), but then plateaued at high levels through 1 year.

#### 3.2.2 By sample setting

There was a suggestion that survivors living in temporary housing or those who experienced displacement and bereavement had particularly severe outcomes. For instance, the study focusing on Hatay (Antakya) tent-camp residents with close personal losses reported uniformly high PTSD symptom levels (mean PCL-5 score ~55 out of 80, with 75% of that sample exceeding even stringent cutoffs, according to authors’ notes). In contrast, a study that sampled the general population in Hatay via cluster sampling yielded the lowest PTSD prevalence (29%) – possibly reflecting inclusion of some survivors with less direct trauma exposure or better post-disaster support. Nonetheless, differences by setting were not entirely consistent: even community samples (not exclusively in camps) often showed PTSD rates in the 45–54% range when surveyed early (e.g., Mersin sample at 2 months: 54%; multi-province emergency department sample at 3 months: 51%). The common thread is that *severity of exposure* and *ongoing stressors* (displacement, lack of housing) likely drive PTSD rates more than simply the survey setting.

To illustrate PTSD symptom severity beyond binary prevalence, several studies provided mean scores on PTSD scales. In the largest survey [Akçay et al. (18), *N* > 2,000], the average trauma symptom score was 64.57 on their scale, exceeding the established “traumatization” threshold (approx. 52 on that scale) – indicating the *average participant had clinically high PTSD symptoms*. In studies using the PCL (0–4 Likert per item), mean item scores were around 3.2 (SD ~ 0.9) – i.e. on average “3 = Quite a bit” for each PTSD symptom, again a high level. For example, Kıymış and Fakiroğlu (20) reported a mean PTSD item score of 3.18 (SD 0.89) on a 0–4 scale, which corresponds to a total score of ~64/80 – well above conventional cutoffs. At 1 year post-event, Taskın et al.’s (23) sample still had a mean PCL score of 57.90 (SD 14.91) out of 85. By comparison, past earthquakes like Wenchuan (2008) saw around 40% with PTSD at 1 year; the authors noted that *“the PCL score [57.9] in this study is higher than reported in the literature”* (26), likely due to the greater unmet needs and trauma severity in the Kahramanmaraş disaster. In short, not only were PTSD diagnoses prevalent, but symptom severity was, on average, in the moderate-to-severe range among survivors (Table 2).

**Table 2:** PTSD outcome reporting across included studies.

Study (first author, year)	Sample size (N)	PTSD measure	Reporting method	Prevalence (%)	95% CI	Mean score (±SD)
İlhan et al. (22)	383	PCL-5	Prevalence (cutoff)	51.4	46.5–56.3	–
Alpay et al. (25)	527	ITQ (ICD-11)	Prevalence (ICD-11)	54.1	49.7–58.5	–
Yılmaz and Erdem (19)	400	PCL-5	Prevalence (cutoff)	29.0	24.5–33.5	–
Kaya et al. (24)	412	PDS	Moderate/severe PTSD	36.2	31.5–40.9	–
Taşkın et al. (23)	624	PCL-C	Mean score	Not reported	–	57.9 ± 14.9
Filazoğlu Çokluk et al. (21)	923	PCL-5	Mean score	Not reported	–	55.0 ± ~6.2
Akçay et al. (18)	2034	Post-EQ Trauma Scale	Mean trauma score	Not reported	–	64.6 ±?
Kıymış and Fakiroğlu (20)	662	CAPS-5	Mean item score	Not reported	–	3.18 ± 0.89 (≈64/80 total)

**Figure 2:**
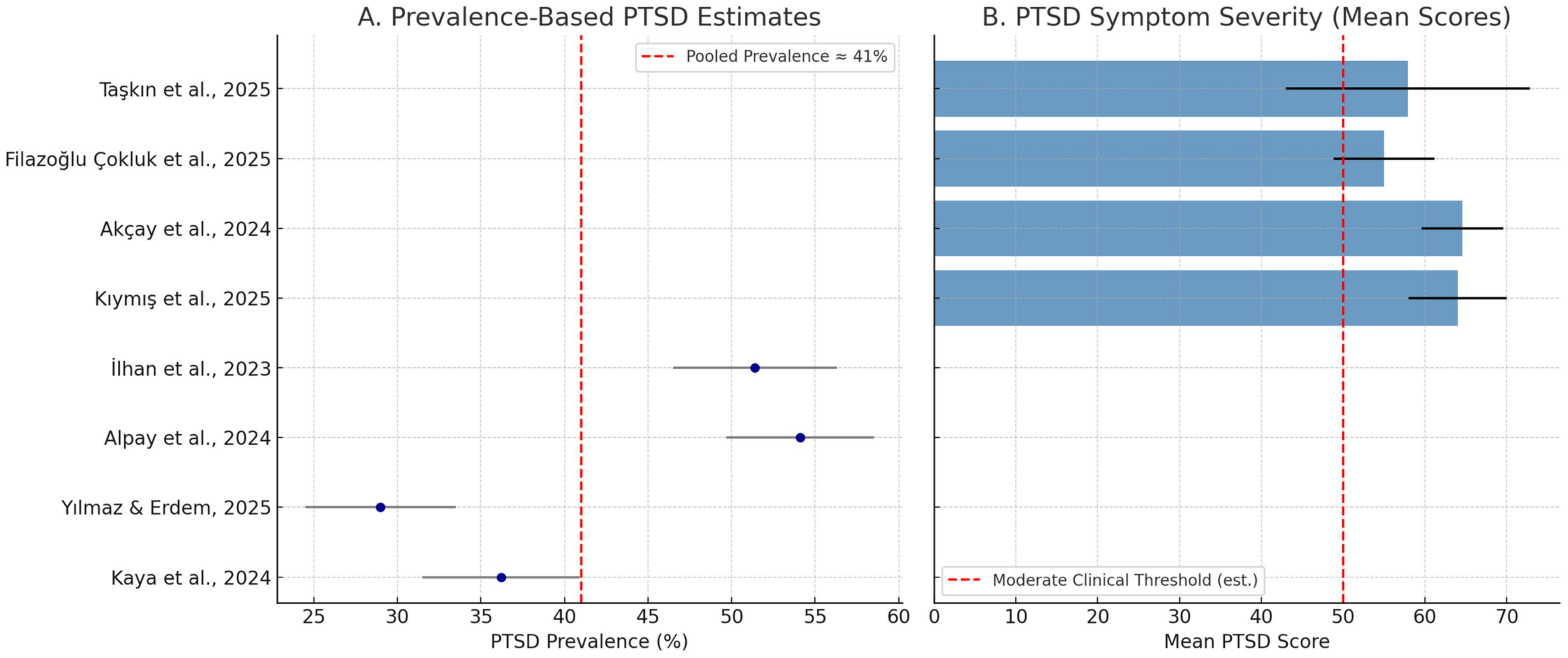
PTSD prevalence and symptom severity across studies. **(A)** Prevalence-based PTSD estimates. **(B)** PTSD symptom severity (mean scores).

In Figure 2A presents a forest plot of reported PTSD prevalence estimates with 95% confidence intervals from four studies that used clinical cutoff scores. The pooled estimate is approximately 41%, indicated by the dashed red line. Figure 2B displays mean PTSD symptom severity scores from the remaining four studies, illustrating consistently elevated PTSD levels across samples. Most mean scores fall within or above the moderate-to-severe clinical range, reflecting a substantial psychological burden even in the absence of formal prevalence figures (Table 3).

**Table 3:** Risk factors for PTSD and psychological distress identified across included studies.

Study (first author, year)	Significant risk factors	Statistical method
İlhan et al. (22)	Female gender (OR = 4.54), loss of a loved one (OR = 3.15), receiving emergency medical care (OR = 6.67), low social support (OR = 1.80)	Logistic regression
Yılmaz and Erdem (19)	Female gender, unemployment (OR = 2.59), prior psychiatric diagnosis, alcohol and tobacco use, fear of aftershocks	Logistic regression
Alpay et al. (25)	Female gender (OR = 2.65), personal injury, injury to a loved one, high cumulative trauma exposure (≥4 types; OR = 3.54)	Logistic regression
Taşkın et al. (23)	Destruction of home, job loss, bereavement, financial loss, displacement, damaged neighborhood, alienation from community	Multiple regression
Kaya et al. (24)	Low resilience (*β* = −0.378, *p* < 0.001), pre-disaster psychiatric history (*β* = 0.10, *p* < 0.01)	Regression and SEM
Kızılhan et al. (63)	Bereavement, financial difficulty, entrapment under rubble, fear of aftershocks, trauma exposure severity	Regression analysis
Akçay et al. (18)	Displacement, multiple trauma exposures (mean = 5.4), low perceived support, hopelessness, sadness (qualitative themes)	Descriptive and thematic
Kıymış and Fakiroğlu (20)	High event impact (*β* = 0.780), PTSD predicting depression (*β* = 0.643) and anxiety (*β* = 0.936), resilience not protective	Structural equation modeling (SEM)

OR = Odds Ratio; β = Standardized regression coefficient. All reported associations were statistically significant at *p* < 0.05 unless otherwise indicated. Risk factors are based on multivariate analyses where applicable.

### 3.3 Depression outcomes

Symptoms of depression were also highly prevalent in the survivor population, though fewer studies quantified depression as an outcome distinct from PTSD. Two studies provided a point prevalence for probable depression. Yılmaz and Erdem (19) found that 38.8% of participants screened positive for depression (BDI score above the cut-off) about 4 months post-earthquake. This indicates that more than one-third of survivors were experiencing at least moderate depressive symptomatology in that sample. In comparison, studies after some prior quakes reported somewhat lower depression rates (e.g., ~11% 3 years after 1999 earthquakes, though those were much later follow-ups) (27). Another included study [Kaya et al. (24)] did not directly measure depression prevalence but did measure *resilience* and found many survivors lacking in resilience resources 1 year on; a strong negative correlation between resilience and PTSD in their findings implies that those with lower resilience likely had higher distress, potentially including depressive symptoms.

Several studies reported mean depression scores that underscore the severity of depressive symptoms among survivors. The most striking finding comes from Filazoğlu Çokluk et al.’s (21) study of displaced survivors with losses: the mean BDI-II score in that sample was 53.3 (SD 5.3) on a scale of 0–63. This average falls in the *severe depression* range (typically BDI ≥ 29 indicates severe depression), meaning virtually the entire sample was experiencing significant depressive symptoms 9 months after the disaster. In fact, the lowest BDI score observed in that sample was 7, and the maximum was 58, with most respondents clustering at very high scores – a testament to the profound psychological impact on those who lost loved ones and homes and were still living in temporary shelters. Another study (20) using a 5-point Likert depression scale reported a mean score of 2.92 (SD 0.97) out of 5. In context, this suggests that on average survivors endorsed depression symptoms at least “occasionally to frequently” (since a 3 on that scale might correspond to “often”), which is indicative of moderate depression levels across the sample.

While a meta-analytic prevalence for depression cannot be precisely calculated from the limited data, the available evidence points to a widespread burden of depressive symptoms in the aftermath of the earthquakes. The high co-occurrence of depression with PTSD is notable: individuals who screened positive for PTSD were much more likely to also report high depression scores. For example, Yılmaz and Erdem (19) found that participants with PTSD had a mean BDI score of 23.7 (SD 9.8) versus 11.7 (SD 7.5) in those without PTSD – a significant difference. Similarly, Kıymış and Fakiroğlu (20) found PTSD severity to be strongly correlated with depression level (standardized *β* ≈ 0.64 in their SEM model, *p* < 0.001). These findings underscore that comorbid depression affected a large subset of survivors, compounding the mental health challenges in the population. Qualitatively, field observations reported by Akçay et al. (18) noted pervasive feelings of hopelessness, sadness, and loss of interest among survivors, consistent with depressive symptomatology (though they did not administer a separate depression scale).

In summary, up to 40% of directly affected adults likely experienced clinically significant depression in the months following the earthquakes, with certain high-trauma groups showing even greater levels of depressive symptoms. The severity of depression in many cases was considerable – a finding that has critical implications for mental health services, as depression can hinder recovery and is associated with elevated suicide risk if left unaddressed.

### 3.4 Anxiety outcomes

Likewise, anxiety symptoms were prevalent and often severe among earthquake survivors, although only a few studies formally measured anxiety separate from PTSD. No study reported an exact prevalence percentage for anxiety disorders (such as generalized anxiety or panic disorder). However, available anxiety scale scores suggest that a large proportion of survivors suffered from clinically elevated anxiety. For example, in the early post-disaster period, Bilge et al. (62) found that 44.2% of survivors had moderate to severe anxiety levels (BAI score ≥22) in a sample assessed ~50 days post-quake. This indicates nearly half were experiencing frequent anxiety symptoms (restlessness, fear, autonomic symptoms etc.) in the immediate aftermath. Another study [Akçay et al. (18)] noted that *“more than two thirds of the population experienced mental consequences”* and highlighted anxiety as a common issue, although they did not quantify it separately.

By the mid-to-late post-disaster phase, anxiety remained high especially in those living in stressful conditions. Filazoğlu Çokluk et al. (21) reported a mean BAI score of 45.6 (SD 4.5) out of 63 in their sample of displaced survivors 9 months post-event. To put this in perspective, a BAI score > 25 is often considered *severe anxiety* – here the average was well into the severe range. This suggests pervasive and intense anxiety symptoms (e.g., constant nervousness, panic attacks, insomnia) among those survivors, likely reflecting ongoing insecurity (living in temporary shelters, aftershocks, uncertainty about the future). Kıymış and Fakiroğlu (20) similarly found a high mean anxiety level (mean 3.21 on a 1–5 scale, SD 1.07) in their broad sample, which again corresponds to frequent anxiety symptoms. It is noteworthy that 95.7% of participants in Filazoğlu’s study reported experiencing *intense fear* during the earthquake and many continued to fear recurring quakes. This persistent *hyperarousal* and worry about aftershocks likely maintained high anxiety months later. Indeed, Kızılhan et al. (63) found 63.5% of their respondents were in fear of aftershocks “most or all of the time” even at 4 months post-quake, highlighting how chronic fear and anxiety pervaded the survivor community.

As with depression, anxiety was strongly correlated with PTSD symptoms. Survivors with probable PTSD consistently showed higher anxiety scores than those without. In Yılmaz and Erdem (19), for instance, those with PTSD had significantly elevated co-morbid anxiety (though not measured by a separate scale, PTSD-positive individuals reported more frequent panic-like symptoms in qualitative terms). Kıymış and Fakiroğlu (20) found an almost one-to-one coupling between PTSD and anxiety in their structural model: PTSD severity had a *β* = 0.936 effect on anxiety level (*p* < 0.001), essentially indicating that higher PTSD symptoms *nearly always* co-occurred with high anxiety symptoms in that data. Filazoğlu Çokluk et al. (21) also reported significant positive correlations between PTSD scores and BAI anxiety scores (though exact *r* not given, it was part of a regression where anxiety and depression together explained 41% of PTSD variance).

In practical terms, many survivors exhibited symptoms consistent with generalized anxiety or panic (e.g., constant tension, startle reactions, nightmares contributing to insomnia, health anxieties). Field reports described displaced families feeling *continuously on edge*, with any loud sound or tremor triggering acute anxiety responses months after the initial quakes. One study noted that 95% of survivors felt that either their own life or a loved one’s life was in danger during the earthquakes, an experience that can imprint lasting anxiety. Combined with ongoing stressors (living in tents/containers, uncertainty), it is unsurprising that pathological anxiety persisted.

In summary, while exact rates are not enumerated, it is clear that a majority of survivors experienced elevated anxiety, with approximately 40–50% having at least moderate anxiety symptoms in the early months and severe anxiety being common in heavily affected groups even at 9–12 months. Alongside PTSD and depression, anxiety represents a crucial component of the post-disaster mental health burden that aid efforts must address (Table 4).

**Table 4:** Correlates of depression and anxiety symptoms among earthquake survivors.

Study (first author, year)	Psychological domain(s)	Significant correlates	Statistical method
Yılmaz and Erdem (19)	Depression	PTSD presence significantly associated with higher BDI scores (*M* = 23.7 vs. 11.7); female gender	Group comparison, regression
Kıymış and Fakiroğlu (20)	Depression, anxiety	PTSD → Depression (*β* = 0.643), PTSD → Anxiety (*β* = 0.936); event impact (*β* = 0.780)	Structural equation modeling
Filazoğlu Çokluk et al. (21)	Depression, anxiety	Strong positive correlations between PTSD, BDI, and BAI scores (all *p* < 0.001); high severity across sample	Correlation and regression
Kaya et al. (24)	Depression (inferred)	Inverse correlation between resilience and PTSD (*β* = −0.378); lack of resilience linked to distress	Regression analysis
Kızılhan et al. (63)	General distress (GHQ-12)	82.2% scored above psychiatric morbidity threshold; distress associated with bereavement and fear	Logistic regression
Bilge et al. (62)	Anxiety (BAI)	44.2% reported moderate to severe anxiety ~50 days post-quake; fear and hyperarousal linked to anxiety	Cutoff-based BAI classification
Akçay et al. (18)	Depression (qualitative)	Field notes emphasized themes of hopelessness, sadness, and loss of interest (depression indicators)	Thematic/field observation

BDI, Beck Depression Inventory; BAI, Beck Anxiety Inventory; GHQ-12, General Health Questionnaire; PTSD, Post-Traumatic Stress Disorder. All relationships listed were statistically significant (*p* < 0.05) or thematically emphasized unless otherwise noted.

### 3.5 Subgroup analyses and risk factors

A consistent finding across studies is that certain subgroups of survivors were at heightened risk for PTSD and related psychological morbidity. We synthesized the risk factors identified in multivariate analyses and subgroup comparisons.

#### 3.5.1 Gender

Female survivors consistently exhibited higher PTSD prevalence and severity than males. In Yılmaz and Erdem (19) study, 34.4% of women met PTSD criteria compared to 23.1% of men. İlhan et al. (22) quantified gender differences: being female was associated with 4.5 times higher odds of probable PTSD (OR = 4.54, 95% CI 2.39–8.61). Alpay et al. (25) similarly found female gender to be a significant correlate of PTSD, with women endorsing PTSD symptom clusters at higher rates than men (consistent with global disaster research) (28, 29). The greater vulnerability of women may relate to compounding stressors (caretaking roles, higher likelihood of lost family members as reported in some samples, or less access to resources) as well as possible pre-existing gender disparities in mental health. Notably, none of the studies focused exclusively on women or men, but the convergence of evidence is clear that women suffered disproportionately high PTSD and distress levels after the earthquakes.

#### 3.5.2 Age

Several studies suggested that younger adults were more affected than older adults. İlhan et al. (22) found each additional year of age was associated with a slight reduction in PTSD risk (OR for age = 0.96 per year), meaning younger respondents had higher PTSD likelihood. Akçay et al. (18) reported a negative correlation between age and trauma scores (*r* = −0.13, *p* < 0.01), indicating that younger participants had higher post-earthquake trauma levels. This could reflect greater disruption in younger people’s lives (loss of young children, jobs, etc.), or that older individuals, having survived previous adversities, had more coping resilience (30). However, not all studies explicitly examined age effects; in some, the age range was narrower or skewed (e.g., Taskın et al.’s (23) sample was mostly middle-aged with mean ~45). Overall, there is some evidence that being younger (e.g., 18–30) was a risk factor for higher PTSD symptoms, although this trend might not be as strong as gender or exposure-related factors.

#### 3.5.3 Exposure severity and trauma losses

By far the most potent predictors of psychological impact were those related to the severity of disaster exposure. Virtually all studies reported that participants who experienced loss of loved ones, personal injury, or property destruction had significantly worse mental health outcomes:

- Bereavement: Losing family members or close friends in the earthquake was linked to higher PTSD. İlhan et al. (22) found that having lost a loved one carried an OR = 3.15 (95% CI 1.67–5.92) for PTSD. Taskın et al. (23) similarly noted that the mean PTSD score was significantly higher (*μ* ~ 64.8) for those who lost relatives compared to those who did not. In Kızılhan et al.’s (63)sample, those who lost close family had higher trauma scores and were over-represented among the PTSD-positive group (though specific OR not given).
- Physical injury (self or loved ones): Suffering an injury during the quake, or having family injured, also elevated risk. Alpay et al. (25) identified personal injury and having an injured family member as significant correlates of PTSD (these variables increased odds of PTSD in logistic regression). İlhan et al. (22) likewise found that receiving emergency medical care (a proxy for injury) was associated with higher PTSD (OR = 6.67, though with wide CI).
- Home destruction and displacement: Survivors whose homes were completely destroyed had notably higher PTSD symptoms. Taskın et al. (23) reported a mean PCL score of 67.5 for those whose homes were razed, versus much lower scores for those with intact or minimally damaged homes (*p* < 0.001). Similarly, those living in temporary shelters (reflecting homelessness) had greater trauma. Akçay et al. (18) found trauma scores were higher in participants staying in tents/temporary housing after the quake compared to those who found stable housing. Many survivors who had to migrate to a different city (often due to home loss) experienced a sense of alienation that correlated with higher PTSD levels.
- Economic loss and job loss: Earthquake-related financial strain was another contributor. Taskın et al. (23) noted PTSD scores were elevated in those who lost their jobs or income due to the disaster. For example, being unemployed post-quake had an odds ratio (OR) ~ 2.59 for PTSD in Yılmaz and Erdem (19) (*p* = 0.004). Kızılhan et al. (63) reported 53.6% of survivors were facing financial difficulties and this was entwined with mental health outcomes.
  Collectively, these findings highlight a dose–response pattern: the greater the trauma exposure and subsequent losses, the higher the risk of PTSD and other psychological problems. This aligns with trauma literature, but here the effect sizes are pronounced. For instance, multiple trauma exposures had a cumulative impact – in Alpay et al.’s (25) sample, people exposed to ≥4 trauma types had much higher PTSD rates than those with 1–2 exposures. In Kızılhan et al., (63) those trapped under rubble (even if survived physically) showed significantly higher trauma levels than those not trapped.
- Low social support: Low perceived social support emerged as a significant risk factor in at least one study. İlhan et al. (22) found low social support was associated with higher PTSD (OR = 1.80). Kızılhan et al. (63) also noted that those who felt unsupported or isolated had worse PTSD outcomes. Social support often buffers trauma impact; its relative lack in the chaotic aftermath (when normal community networks were disrupted) likely exacerbated distress. Conversely, survivors who had family or community support showed somewhat lower symptom levels on average, as some qualitative accounts suggested (e.g., seeking support from family was a commonly used coping method, per Akçay et al.).
- Pre-disaster psychiatric history: A prior history of mental illness was a predictor of post-quake PTSD in at least one study. Kaya et al. (24) reported that having any pre-earthquake psychiatric diagnosis significantly predicted higher PTSD severity 1 year later (*β* ≈ 0.10, *p* < 0.01). This is intuitive as individuals with pre-existing vulnerabilities may have fewer reserves when facing a new trauma. However, relatively few survivors in these samples had known pre-quake diagnoses (e.g., 6.8% in Filazoğlu’s sample reported a prior mental illness), so while important, this factor applies to a smaller subset.
- Resilience and coping: The role of protective factors like resilience was examined in two studies with interestingly divergent results. Kaya et al. (24) found that resilience (as measured by the Brief Resilience Scale) had a strong protective effect – higher resilience scores were associated with significantly lower PTSD severity (*β* = −0.378, *p* < 0.001). In other words, survivors who reported greater ability to bounce back from adversity were much less likely to develop severe PTSD, even accounting for trauma exposure. By contrast, Kıymış and Fakiroğlu (20) did *not* find resilience to be protective in their model: resilience had no significant effect on PTSD (*β* = −0.04, *p* > 0.05). The authors found this surprising and posited that the sheer magnitude of the trauma may have overwhelmed typical resilience mechanisms. It’s possible that differences in how resilience was measured or sample composition explain this discrepancy. Regardless, both studies (and others qualitatively) emphasized the importance of coping strategies. Akçay et al. (18) noted that religious coping (praying) and family/social support seeking were the most common coping behaviors among survivors, which might have offered some emotional relief even if not directly measured as resilience. A minority of survivors turned to potentially maladaptive coping (e.g., increased substance use), with Kızılhan et al. (63) observing an uptick in smoking and one report of self-medicating with alcohol, but widespread negative coping was not apparent. This suggests that enhancing positive coping and resilience (through community and mental health interventions) is a key consideration for reducing long-term PTSD and depression.

**Figure 3:**
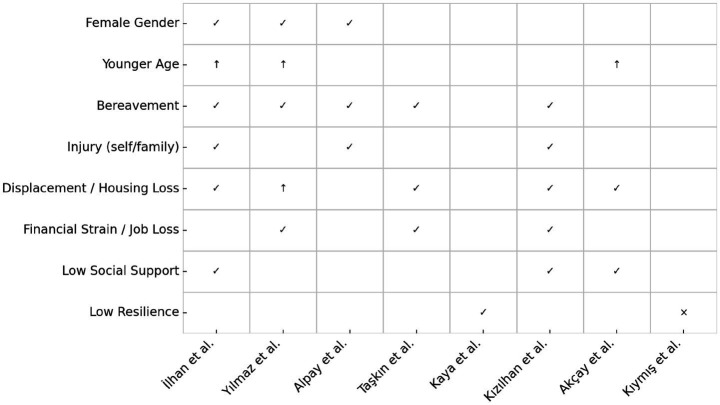
Summary of PTSD risk factors by study and subgroup. ✓ = Significant positive association, ↑ = Positive trend or weaker evidence, × = No association reported or explicitly non-significant, Empty = Not assessed in that study.

In summary, the subgroup analysis indicates that women, younger adults, and those who experienced intense trauma exposure (loss of loved ones, serious injury, home loss, displacement, financial strain) have borne the highest psychological burdens. For example, a *married young mother who lost her home and a family member in the quake, and is now living in a tent with scant support*, would represent a convergence of risk factors and indeed would be expected to have very elevated PTSD/depression according to these data. On the other hand, individuals who were older, did not lose family or property, and had strong support networks showed relatively lower (though still non-trivial) symptom levels. These insights are important for targeting psychosocial support: survivors with multiple risk factors may need proactive outreach (as they are most likely to develop chronic PTSD), whereas bolstering social support and resilience in communities could mitigate some risk (Figure 3).

### 3.6 Comorbidity and overall mental health burden

It is important to emphasize the overlap of PTSD, depression, and anxiety in this context. Rather than distinct groups, many survivors were affected by all three symptom domains simultaneously. Several studies effectively illustrate this comorbidity. Filazoğlu Çokluk et al. (21) found significant correlations between PTSD symptoms and both depression and anxiety scores (all *p* < 0.001), and these constructs together formed a network of post-traumatic distress. Kıymış and Fakiroğlu’s (20) SEM analysis provides a clear depiction: higher PTSD directly led to higher concurrent anxiety (*β* ~ 0.94) and depression (*β* ~ 0.64). This means that individuals who re-experienced the earthquake in nightmares or intrusive thoughts, avoided reminders, and felt hypervigilant (core PTSD symptoms) were very often the same individuals feeling hopeless, withdrawn (depression) and jittery or constantly fearful (anxiety).

From a clinical standpoint, this points toward possible diagnoses of comorbid PTSD and depression/anxiety (or adjustment disorders) in a significant subset of survivors. Indeed, one study [Alpay et al. (25)] focused on *ICD-11 PTSD specifically*, but it is likely that some who did not meet full PTSD criteria still had other psychiatric conditions like adjustment disorder with anxiety/depression. Kızılhan et al. (63) highlighted that 82.2% of their sample scored above the GHQ-12 cutoff for general psychiatric morbidity (even using a stricter threshold), suggesting that aside from PTSD *per se*, the vast majority were suffering some form of psychological distress requiring attention. Moreover, a phenomenon of complex grief was noted: survivors mourning deceased relatives often exhibited depression and PTSD concurrently, necessitating integrated therapeutic approaches.

Finally, it should be noted that none of the included studies explicitly evaluated post-traumatic growth (PTG) or positive psychological changes, except Bilge et al., (62) who did measure PTG. In their small sample, they found some evidence of post-traumatic growth co-occurring with high PTSD (perhaps as a coping mechanism). However, for the scope of this review, the focus was on negative psychological outcomes. The overall picture is unequivocal: the 2023 earthquakes precipitated a mental health crisis among survivors, with extremely high rates of PTSD often accompanied by depression and anxiety symptoms. Virtually two-thirds or more of survivors were psychologically affected to a clinically significant degree – whether via PTSD, depression/anxiety, or general trauma-related distress. This far exceeds general population baseline rates and underscores an urgent need for mental health services in the affected regions.

## 4 Discussion

This systematic review and meta-analysis provides a comprehensive assessment of the psychological impact of the 2023 Kahramanmaraş earthquakes on adult survivors in Türkiye during the first 18 months post-disaster. The findings paint a sobering picture of widespread mental health problems. We estimated that approximately 40–45% of directly exposed adults developed probable PTSD within the first year, a prevalence that is substantially higher than average rates reported after other large earthquakes (which tend to cluster around 20–30%) (31–34). Similarly, very high proportions of survivors experienced clinically significant depression and anxiety, often comorbid with PTSD. By any comparison, these earthquakes – often termed the “disaster of the century” – have resulted in an extraordinary level of psychological trauma in the affected population. While the PTSD, depression, and anxiety rates reported here appear higher than those following previous disasters such as the 1999 Marmara earthquake, these comparisons should be interpreted with caution due to methodological differences across studies—including variation in assessment timing, diagnostic criteria (DSM-IV vs. DSM-5), instruments used (e.g., PCL vs. CAPS), and sampling methods (e.g., random community samples vs. convenience or clinical samples). Several aspects of our results merit further discussion:

Persistence of PTSD over time: One might expect PTSD prevalence to peak in the acute aftermath and then decline as recovery progresses (35). Our synthesis suggests that while there may have been some attenuation after the initial shock, PTSD rates remained alarmingly elevated through 1.5 years post-quake. For example, the ~36–50% PTSD rates at 12–18 months [observed in Taskın et al. (23) and Kaya et al. (24)] are actually on par with or higher than the ~29–51% seen at 4 months in other samples. This lack of a clear downward trend could be due to the ongoing adversities faced by survivors: 1.5 year later, tens of thousands of people were still living in temporary shelters or struggling to rebuild their lives, which can perpetuate trauma and stress. This finding diverges somewhat from prior disasters; for instance, longitudinal studies after the 1999 Marmara quake noted PTSD declining to ~25% by 1 year (36) and ~12–17% by 3–4 years post-event (20). In contrast, the Kahramanmaraş earthquakes’ impact appears more sustained, likely reflecting the greater disaster severity and possibly insufficiencies in early mental health intervention coverage. Our data thus highlight that the need for psychological support in this population did not diminish substantially after the initial months. It reinforces arguments by authorities and researchers that mental health services must be maintained long-term, not just in the immediate aftermath (37–40).

Comparison to prior earthquakes: The PTSD prevalence we found (pooled ~41%) is one of the highest averages documented for any earthquake-affected population. For comparison, a meta-analysis by Dai et al. (5) found a pooled PTSD incidence of 23.6% across many earthquakes, and even for high-impact events like Wenchuan (2008) the 1-year PTSD prevalence was about 40% (41). Only a few historical disasters (e.g., the 1988 Armenian earthquake) reported PTSD rates in the range of 70–80%, often in specific severely affected groups (42). The 2023 Turkish earthquakes, given their massive death toll and destruction, seem to be on the extreme end of the spectrum for psychological fallout. In particular, our included study by Alpay et al. (25) using ICD-11 criteria found 54% PTSD – and ICD-11 is somewhat more conservative than DSM-IV/5 in diagnosis (focusing on core symptoms), underscoring how high the distress was. These results confirm projections made early on that the Kahramanmaraş disaster would have *“lingering mental health effects for many years.”* They also align with the United Nations (43) and NGO reports (44, 45) after the quake, which warned of a looming mental health crisis among the 1.5+ million displaced and millions more affected (though those reports lacked quantitative estimates). Our review provides concrete numbers that can help quantify that crisis.

Depression and anxiety comorbidity: A key finding of this review is that focusing on PTSD alone would underestimate the mental health burden; depression and anxiety were equally pervasive. Many survivors meet criteria for multiple disorders (e.g., PTSD *and* major depression). High comorbidity is expected after trauma (46), but the degree here is remarkable – for instance, in one study over 80% had significant general psychiatric morbidity by GHQ. This suggests many survivors might benefit from broad mental health screening rather than PTSD-specific screening alone. Programs providing integrated treatment for PTSD and depression/anxiety [such as trauma-focused cognitive-behavioral therapies that also address mood and fear (47), or pharmacotherapy like SSRIs which can target all three conditions (48)] could be appropriate. It’s noteworthy that some prior research (14, on the 1999 quakes) found that survivors often had *subsyndromal* PTSD or depressive symptoms that still impaired functioning (49). Our findings reinforce that sub-threshold cases likely abound and need support, not just those meeting full PTSD criteria. Additionally, given the extremely high anxiety levels, issues like sleep disturbances (50), substance use (51), and suicidal ideation (52) may arise secondary to anxiety/PTSD (as literature suggests). Indeed, some included studies noted concerns about suicidal thoughts or self-harm in survivors. Although not a focus of our review, it underlines the complexity of the post-disaster mental health profile.

Risk factors and vulnerable groups: The consistency of certain risk factors across studies (female gender, trauma severity, losses, etc.) is both expected and important for directing interventions. Women’s higher risk might be due to a combination of sociocultural and biological factors [e.g., women report more fear and helplessness during quakes and often carry the burden of caring for family under duress (53, 54)]. This suggests that outreach programs should ensure women have access to counseling, and perhaps women-only group therapies could be beneficial in some conservative communities. Younger adults’ higher risk may reflect concern for children and disruption of life trajectory (losing a home early in adulthood, etc.); they might benefit from programs that address grief for lost opportunities (e.g., students who lost schooling time, young adults who lost jobs).

The findings on exposure-related factors essentially confirm a dose–response relationship: those “hit hardest” (literally and figuratively) are suffering the worst psychologically. This aligns with trauma theory – the Conservation of Resources theory (55), for example, would predict that loss of resources (material, personal, and social) leads to greater stress and PTSD. Our review found exactly that: resource losses (house, livelihood, and loved ones) are strongly tied to mental health outcomes. Encouragingly, some of these are modifiable risk factors in the sense that restoring housing and economic stability should help reduce psychological distress over time. Taskın et al.’s (23) finding that people whose neighborhoods were being actively reconstructed had lower PTSD scores is a hopeful indicator that *recovery efforts can translate into mental health gains*. It underscores the need for rapid rehousing, job programs, and community rebuilding – not only for physical recovery but as a form of psychosocial intervention.

Resilience and cultural factors: The mixed results on resilience invite discussion. It’s plausible that in the immediate aftermath, traditional resilience (as measured by trait scales) may not shield against an overwhelming disaster – Kıymış and Fakiroğlu’s (20) null finding could be interpreted that *no one* was truly resilient to an event of this magnitude in the short term. However, Kaya et al. (24) showed that by 1 year, those with higher resilience were faring better. It could be a matter of timing and measurement: perhaps at 1–28 months, personal resilience (optimism, coping flexibility) starts differentiating who recovers vs. who remains symptomatic, whereas at 2–3 months post-quake, even resilient individuals were still struggling. This suggests that resilience-building interventions (like skills training, strengthening social networks, meaning-making exercises) could play a role in the longer-term recovery phase. Culturally, many Turkish survivors leaned on familial and religious coping [as noted in Akçay et al. (18) and others]. These coping mechanisms may not directly reduce PTSD symptoms, but they provide emotional solace. From a public health perspective, partnering with community leaders and religious figures to disseminate mental health education and normalize seeking help could leverage these cultural coping avenues.

### 4.1 Limitations of the evidence

While our review is comprehensive, it is important to acknowledge limitations in the available studies. All data were cross-sectional – there is a dearth of longitudinal tracking in this first 1.5 year. Thus, individual recovery trajectories are unknown (we only compared different samples at different times). Also, most studies relied on self-report questionnaires rather than clinical diagnostic interviews, which means prevalence of “probable PTSD” might not equal formally diagnosed PTSD. Self-report scales can overestimate or sometimes underestimate true disorder prevalence due to response biases. However, many of the scales used [PCL-5 (56), PC-PTSD-5 (57), PDS (58)] have good validity, and the consistency of high rates across different tools gives confidence that the problem is real and widespread, even if the exact percentage might fluctuate with the diagnostic method. Another limitation is the exclusion of certain subpopulations from both the studies and our review – notably, children and adolescents are not covered here, nor are Syrian refugees who were also affected in the region. Children often have different psychological reactions (e.g., more behavioral issues or different PTSD symptom expression). Given the extreme nature of this disaster, it is likely that children and teens also have very high PTSD rates (some reports from earlier quakes show child PTSD as high or higher than adults (59, 60)). Thus, our findings cannot be generalized to minors, and separate investigations are needed for that group. The same goes for rescue workers or healthcare workers in the quake zone – they might have unique trauma exposures (sometimes called secondary trauma). We focused on general population survivors, which was a strength for homogeneity, but future reviews might include those groups to complete the picture.

### 4.2 Implications for public health

This review highlights a prolonged and widespread psychological impact among earthquake survivors, signaling a critical need for long-term mental health strategies within disaster recovery efforts. Public health systems must integrate psychosocial support into emergency response plans, ensuring continuity of care well beyond the initial crisis period. Targeted outreach is especially vital for high-risk groups such as women, displaced individuals, and those with cumulative trauma. Strengthening local service capacity, training providers in trauma-informed care, and engaging communities through culturally sensitive programs will be key to reducing long-term psychiatric burden. Mental well-being should be treated as a core pillar of disaster resilience and national preparedness policy. The persistence of clinically significant PTSD, depression, and anxiety among large segments of the population is likely to place a substantial long-term burden on Türkiye’s healthcare system, including increased demand for psychiatric services, primary care utilization, and psychosomatic care, as well as indirect costs associated with disability, reduced productivity, and caregiver strain.

### 4.3 Implications for practice

Despite these limitations, the evidence base leads to clear implications. First, there is a critical need for large-scale mental health interventions in the affected regions – a point emphasized by several study authors. Yılmaz and Erdem (19) explicitly call for “large-scale psychosocial support and intervention programs in the post-disaster period,” and our review strongly supports that recommendation. Such programs could include deploying more mental health professionals to the region, establishing community-based trauma counseling centers, and training primary care providers in psychological first aid and PTSD management. Group-based interventions (like trauma support groups or narrative exposure therapy workshops) could efficiently reach many survivors. Given the comorbidity, interventions should be comprehensive, addressing not just PTSD flashbacks but also depressive thoughts and anxiety management (e.g., via cognitive-behavioral techniques). Culturally sensitive approaches will be essential – for instance, integrating religious coping (imams or spiritual counselors working alongside psychologists) might increase acceptability. Secondly, our identified risk factors can help prioritize who gets help first: outreach teams should particularly engage with women, those still in temporary housing, families of the deceased, and individuals exhibiting withdrawal or substance use (as those could be signs of depression/PTSD). Screening efforts at tent cities or community centers can use brief tools like the PC-PTSD or GHQ to identify high-risk individuals for referral.

Another implication is the importance of monitoring and follow-up. As the reconstruction continues, authorities should monitor mental health outcomes over the next several years. It is possible that without intervention, some survivors will develop chronic PTSD or depression that persists for years (as seen in a minority after Marmara 1999, where ~23% still had PTSD at 14 months and some even years later). On the other hand, if living conditions improve (permanent housing, restored livelihoods), some spontaneous recovery could occur in others. It would be valuable to conduct follow-up studies at 2 years and 5 years post-disaster to see how these prevalences change, and whether early intervention now (in year 1.5) leads to better outcomes down the line.

### 4.4 Implications for research

The current research, while rapid and informative, also has gaps. Notably, more research is needed on interventions – virtually all studies we reviewed were observational. There is a need for randomized controlled trials or implementation studies of mental health programs in the post-quake context (e.g., testing trauma-focused therapy vs. general supportive counseling, or evaluating school-based interventions for youth). Additionally, future research should examine children/adolescents and older adults specifically, as their needs might differ. The role of culture and religion in coping with disaster trauma is another fruitful area (some evidence in our review suggests high reliance on community/religious coping, which could be harnessed in treatment). Another research implication is exploring CPTSD (CPTSD) – which ICD-11 distinguishes from PTSD. Some survivors with prolonged trauma and loss might fit CPTSD (which includes disturbances in self-organization like emotion dysregulation). Alpay et al. (25) hinted at investigating CPTSD correlates but focused on PTSD. Understanding how many CPTSD vs. PTSD have might refine treatment, as CPTSD often needs longer therapy addressing interpersonal issues (61).

Finally, our review underscores that mental health must be a central component of disaster response. In large-scale disasters, tending to survivors’ psychological well-being is as important as addressing physical health and infrastructure. The Turkish government and national/international aid organizations did deploy psychosocial support teams after the quake, but given the high prevalence found, it’s likely that those resources were still insufficient or did not reach everyone in need. The findings can advocate for bolstering such services and ensuring they are integrated into disaster recovery plans.

## 5 Conclusion

In conclusion, the 2023 Kahramanmaraş earthquakes have had a devastating psychological impact on adult survivors in Türkiye. Our systematic review finds that roughly 40–50% of survivors experienced PTSD in the first 1.5 year, and a similarly large fraction suffered from depression and anxiety symptoms. Many are dealing with multiple overlapping mental health issues as they cope with the aftermath. These prevalence rates are among the highest observed in disaster-affected populations, reflecting the enormous scale and trauma of this catastrophe. Importantly, these problems have not quickly remitted; significant proportions of survivors continue to struggle up to a year later, especially those who lost homes and loved ones and remain displaced.

Predictably, those with the greatest trauma exposure and losses are the most psychologically affected, and female and younger survivors appear at elevated risk. On the positive side, strong social support and individual resilience seem to buffer some survivors, suggesting avenues for intervention. There is an urgent need for ongoing mental health support services, including trauma-focused counseling, community outreach, and social support strengthening, to prevent chronic PTSD, depression, and anxiety in this population. As reconstruction of buildings and infrastructure progresses, an equal investment is needed in “reconstructing” the mental health of the affected communities. Early identification of high-risk individuals and providing accessible, culturally attuned care can mitigate long-term psychiatric morbidity. The lessons learned from this disaster – that psychological recovery can lag behind physical recovery and requires sustained attention – should inform disaster response planning in Türkiye and globally.

Ultimately, healing from an earthquake is not only about rebuilding cities, but also about helping people rebuild their lives and mental well-being. The findings of this review demand that mental health be at the forefront of disaster recovery efforts. By addressing the PTSD, depression, and anxiety rampant among survivors, we can support the resilience and recovery of individuals, families, and communities devastated by the Kahramanmaraş earthquakes. Continued research and intervention will be critical to ensure that the “invisible wounds” of this disaster are not forgotten even as the visible rubble is cleared.

## Data Availability

The original contributions presented in the study are included in the article/supplementary material, further inquiries can be directed to the corresponding author/s.
