# Supplementary material for: Psychological impact of the 2023 Kahramanmaraş earthquakes: a systematic review and meta-analysis of PTSD, depression, and anxiety among Turkish adults
Source: Front Public Health. 2025 Aug 26;13:1664212. doi: 10.3389/fpubh.2025.1664212 (PMC12417135; doi:10.3389/fpubh.2025.1664212)
Supplement: Supplementary file 2 [file Data_Sheet_2.docx]

**Full Search Strategy**

**Search Date: May 30, 2025**

**Databases:** PubMed, Scopus, Web of Science

**Search Period:** February 6, 2023 – May 30, 2025

**Search Language:** English only

**Eligibility Filters:** Peer-reviewed journal articles, human studies, adult general population, quantitative studies, sample size ≥370, Turkish population exposed to the earthquake

1. **PubMed Search Strategy**

("Kahramanmaraş earthquake"[Title/Abstract] OR "2023 Turkey earthquake"[Title/Abstract] OR "Türkiye earthquake"[Title/Abstract] OR "earthquake survivors"[Title/Abstract])

AND

("PTSD"[Title/Abstract] OR "post-traumatic stress"[Title/Abstract] OR "posttraumatic stress"[Title/Abstract] OR "depression"[Title/Abstract] OR "anxiety"[Title/Abstract] OR "mental health"[Title/Abstract])

AND

("Turkish population"[Title/Abstract] OR "Turkey"[Title/Abstract] OR "Türkiye"[Title/Abstract])

Filters applied: Publication date from 2023/02/06 to 2025/05/30; English; Humans; Journal Article

2. **Scopus Search Strategy**

(TITLE-ABS("Kahramanmaraş earthquake") OR TITLE-ABS("2023 Turkey earthquake") OR TITLE-ABS("Türkiye earthquake") OR TITLE-ABS("earthquake survivors"))

AND

(TITLE-ABS("PTSD") OR TITLE-ABS("post-traumatic stress") OR TITLE-ABS("posttraumatic stress") OR TITLE-ABS("depression") OR TITLE-ABS("anxiety") OR TITLE-ABS("mental health"))

AND

(TITLE-ABS("Turkish population") OR TITLE-ABS("Turkey") OR TITLE-ABS("Türkiye"))

AND

(LIMIT-TO(DOCTYPE, "ar"))

AND

(LIMIT-TO(LANGUAGE, "English"))

AND

(PUBYEAR > 2022)

3. **Web of Science Search Strategy**

TS=("Kahramanmaraş earthquake" OR "2023 Turkey earthquake" OR "Türkiye earthquake" OR "earthquake survivors")

AND

TS=("PTSD" OR "post-traumatic stress" OR "posttraumatic stress" OR "depression" OR "anxiety" OR "mental health")

AND

TS=("Turkish population" OR "Turkey" OR "Türkiye")

Refined by:

- DOCUMENT TYPES: (Article)

- LANGUAGES: (English)

- TIMESPAN: 2023-2025

Indexes: SCI-EXPANDED, SSCI, A&HCI, ESCI

**Notes:**

- Boolean operators (AND, OR) were used to combine concept blocks.
- No grey literature, editorials, theses, or conference papers were included.
- Hand-searching of reference lists was also conducted to identify additional eligible articles.
